# An Improved Implementation of Codon Adaptation Index

**Published:** 2007-05-17

**Authors:** Xuhua Xia

**Affiliations:** Department of Biology and Center for Advanced Research in Environmental Genomics, University of Ottawa, 30 Marie Curie, P.O. Box 450, Station A, Ottawa, Ontario, Canada, K1N 6N5

**Keywords:** Codon usage bias, translation elongation, gene expression, tRNA

## Abstract

Codon adaptation index is a widely used index for characterizing gene expression in general and translation efficiency in particular. Current computational implementations have a number of problems leading to various systematic biases. I illustrate these problems and provide a better computer implementation to solve these problems. The improved CAI can predict protein production better than CAI from other commonly used implementations.

## Introduction

The efficiency of translating mRNA to protein depends partially on the coding strategy of an mRNA and is reflected in codon usage bias which is often measured by two classes of indices, one class being codon-specific and the other being gene-specific. A representative of the first class is the relative synonymous codon usage or RSCU (Sharp et al. 1986), and a representative of the second class is the codon adaptation index, or CAI (Sharp and Li, 1987).

Other than CAI, several other indices have been proposed to measure codon usage bias of protein-coding genes. All these indices (including CAI) measure codon usage bias in two ways. One is to measure the deviation of codon usage from random expectation or from equal codon usage. A representative of this type of codon usage indices is the effective number of codons (Wright, 1990) which measures codon usage bias by the deviation of codon usage from equal codon usage.

The other codon usage indices measure codon usage bias by their degree of using translationally favored codons. They differ in how they define translationally favored codons. The frequency of optimal codons, or F_op_ (Ikemura, 1985), defines translationally optimal codons as those forming Watson-Crick base pair with the anticodon of major tRNA species in each codon family. The codon adaptation index (CAI) defines translationally optimal codons as those frequently represented in highly expressed genes. The codon bias index, or CBI (Bennetzen and Hall, 1982) defines translationally favored codons as those not only frequently represented by highly expressed genes but also forming Watson-Crick base pair with the anticodon of major tRNA species. Comparative studies (Coghlan and Wolfe, 2000; Comeron and Aguade, 1998) suggest that CAI is the best in predicting gene expression levels.

CAI has been used extensively in biological research. Other than its primary use for measuring the efficiency of translation elongation, it has been used to study functional conservation of gene expression across different microbial species (Lithwick and Margalit, 2005), to predict protein production (Futcher et al. 1999; Gygi et al. 1999), and to optimize DNA vaccines (Ruiz et al. 2006). CAI has recently been used for detecting lateral gene transfer (Bodilis and Barray, 2006; Carbone et al. 2003; Cortez et al. 2005; Sugaya et al. 2004; Tsirigos and Rigoutsos, 2005a; Tsirigos and Rigoutsos, 2005b), although its accuracy and sensitivity in such detection remain to be evaluated.

CAI of a coding sequence (CDS) is computed from (1) the codon frequencies of the CDS and (2) the codon frequencies of a set of known highly expressed genes (often referred to as the reference set) which is used to generate a column of w values:

(1) $$w_{ij}=\frac{f_{ij.\text{ref}}}{\text{Max}f_{i.\text{ref}}}$$

where f_ij.ref_ is the frequency of codon j in synonymous codon family i, and Maxf_i.ref_ is the maximum codon frequency in synonymous codon family i. For example, if the four alanine codons GCA, GCC, GCG and GCU in the reference set have frequencies 200, 40, 40, and 20, respectively, their associated w values will be 1, 0.2, 0.2 and 0.1, respectively. The codon whose frequency is Maxf_i.ref_ is often referred to as the major codon (whose w is 1), and the other codons are referred to as minor codons. The major codon is assumed to be the translationally optimal codon.

The CAI value of a CDS is computed as:

(2) $$CAI\;=\text{exp}\left(\frac{\sum_{i=1}^{m}\sum_{j=1}^{n_{i}}\left[f_{ij}\;\text{ln}(w_{ij})\right]}{\sum_{i=1}^{m}\sum_{j=1}^{n_{i}}f_{ij}}\right)$$

where m is the number of synonymous codon families, n_i_ is the number of synonymous codons in codon family i, and f_ij_ is the frequency of codon j in codon family i. The exponent is simply a weighted average of ln(w). The maximum CAI value is 1.

## Problems with CAI and Solutions

CAI has three implementation problems. Most published papers use the cai program in EMBOSS (Rice et al. 2000), typically referred to as the EMBOSS.cai program. Another software for computing CAI is the web application called CAI Calculator 2 (Wu et al. 2005). I will use both EMBOSS.cai and CAI Calculator 2 to illustrate implementation problems.

### 

#### Problem when w = 0

This problem often happens when only a few genes are known to be highly expressed, so that the number of codons one can compile from a small number of genes is small, leading to some w values to be zero. For example, the frequently used codon usage table in the EMBOSS compilation Eyeastcai. cut (where ‘.cut’ stands for codon usage table) for the budding yeast contains a number of zeros. In particular, in the CGN (coding for arginine) codon family, there are 43 CGU codons, but no CGG, CGA, or CGC codon.

The overuse of CGU and the avoidance of CGG, CGA and CGC codons in highly expressed genes make sense because the yeast genome contains six tRNA^Arg^ genes all with anticodon ACG forming Watson-Crick base-pairing with the CGU codon, but no other tRNA^Arg^ gene forming Watson-Crick base pairing with the other three CGN codons. The highly expressed genes included in the Eyeastcai. cut file apparently have strong codon usage bias favoring the CGU codon, taking advantage of the six ACG-tRNA^Arg^ genes to facilitate translation of arginine codons. While this illustrates well the codon-anticodon adaptation, it causes practical problems with computing CAI.

Given the 43 CGU codon and no other CGN codon in the reference set, the associated w value is therefore 1 for CGU but 0 for the other three. However, computing CAI requires taking the logarithm of w but there is no logarithm defined for w = 0. Different implementations of CAI typically would try to use some methods to avoid taking the logarithm of 0, but the resulting CAI can be outrageous. For example, if one uses the following sequence consisting of CGA, CGC, CGG codons only:

S = CGACGCCGGCGACGCCGGCGACGCCGGCGACGCCGG

as input to the EMBOSS.cai program (which is available online at http://bioportal.cgb.indiana.edu/cgi-bin/emboss/cai), the resulting CAI value is 1 (the maximum CAI), which is obviously incorrect. We know that, among CGN codons, only CGU is represented in the reference set and all other three CGN codons have zero representation in the reference set. The sequence S consists of only CGA, CGC and CGG codon only but no CGU, and we therefore would expect the CAI to be at its minimum, i.e., 0. A CAI of 1 from EMBOSS.cai for sequence S is of course wrong. A correct implementation should yield a CAI of 0 for S with a warning that there is insufficient information for computing CAI for S.

The output from the web application CAI Calculator 2 (Wu et al. 2005), available at http://www.evolvingcode.net/codon/cai/cais.php, is even more puzzling. If the input sequence is made of two CGC codons only, then CAI is 0, which seems to make sense. However, when the input sequence is made of 4, 8 or 16 CGC codons, respectively, the output CAI becomes 0.001, 0.002 and 0.003, respectively. CAI should depend only on the codon frequencies of the input sequence, not on the absolute number of codons in the input sequence, i.e. it should not increase with increasing sequence lengths.

The original proposal (Sharp and Li, 1987) to solve the problem of w = 0 is to change it to 0.5. This is also not satisfactory because sequence S would then have a CAI = 0.5 instead of 0.

#### Problems with codon families containing a single codon

EMBOSS.cai does not exclude codon families with a single codon in computing CAI. It is important to exclude such codons. Note that, for such codons (e.g. AUG and UGG in the standard genetic code), their corresponding w value will always be 1 regardless of codon usage bias of the gene. If a gene happens to use a high proportion of methionine and tryptophan, then it will have a high CAI value even if its codon usage is not at all biased. Just add a string of AUG triplets to a sequence will substantially increase its CAI. For example, if the input sequence consists of multiple AUG codons, such as

S = AUGAUGAUG......

then the EMBOSS.cai program will yield a CAI value of 1, based on the web interface of EMBOSS. cai. The CAI Calculator 2 also generates a CAI of 1 with this multi-AUG input sequence. Such a CAI value is obviously not warranted. A correctly computed CAI value should exclude codon families each containing a single codon.

The original paper proposing CAI (Sharp and Li, 1987) specifically stated that codon families containing a single codon (e.g. AUG and UGG in the standard genetic code) should be excluded in computing CAI. It is strange that existing software for computing CAI often ignore this statement.

#### Problem with amino acids coded by two separate codon families

EMBOSS.cai and CAI Calculator 2 also produce other perplexing output. Suppose we now use a sequence consisting entirely of CGU codons and expect the resulting CAI to be 1 by using the Eyeastcai.cut reference set (Recall that the reference set contains 43 CGU codons but no CGA, CGC or CGG codon). The resulting CAI value from the EMBOSS.cai program is 0.140 instead of 1. This is again unexpected. It turns out that amino acid arginine is coded by two codon families, the CGN codon family we have mentioned, and the AGR codon family. The largest codon frequency among these six codons is 314 (for AGA codon). So the w value for CGU is not 1 (= 43/43) as we have thought, but is only 0.1369 (= 43/314). For standard genetic code, there are three amino acids (arginine, leucine and serine) each coded by two different codon families. EMBOSS.cai, as well as CAI Calculator 2, does not separate the two codon families for each amino acid, but treated them as three six-member codon families. This is not appropriate because the codon usage bias in one codon family (e.g. the CGN codon family) translated by one set of tRNAs is much obscured by the codon usage in another codon family (e.g. the AGR codon family) translated by another set of tRNA genes. A correct implementation should separates each six-member codon family into two separate codon families, with one family containing two codons and another containing four.

The original paper proposing CAI (Sharp and Li, 1987) did not explicit specify how to treat such six-member codon families, but their equation (8) indicates no separation of such codon families into a two-member and four-member codon families. This is unfortunate.

## User Interface

**Figure 1:**
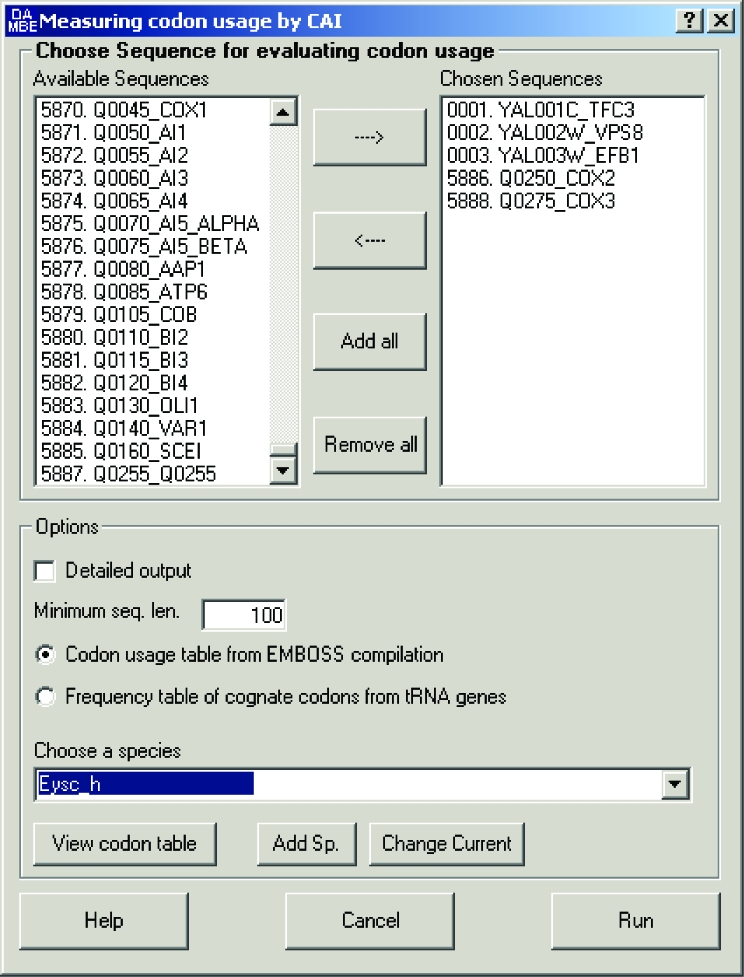
User interface for computing CAI in DAMBE. The top left panel lists the sequences in DAMBE’s buffer (5888 coding sequences from *Saccharomyces cerevisiae* genome). The top right panel lists sequences chosen to compute CAI. Clicking ‘Add all’ will include all sequences for analysis. The set of reference sequences for each species is selected by the dropdown box labeled ‘Choose a species’. The reference codon usage can be viewed by clicking ‘View codon table’. Adding one’s own reference codon usage table is done by clicking the ‘Add sp.’ button.

The improved CAI is implemented as a new function in DAMBE (Xia, 2001; Xia and Xie, 2001, freely available at http://dambe.bio.uottawa.ca/dambe.asp), which uses a windowed user interface (Fig. 1). DAMBE can read 20 standard sequence file formats including files in the simple FASTA format and the more involved GenBank format or trace files from automatic sequencers. The CAI function can be accessed by clicking ‘Seq. Analysis|Codon usage|CAI’. The ensuing dialog box is self-explanatory, except that, for species without a reference set of highly expressed genes, a codon table based on tRNA anticodon can be used by clicking the alternative option button.

## Evaluation of the Improvement

The ultimate test of the utility of a codon usage index such as CAI is whether it can contribute to accurate prediction of protein production. However, CAI reflects (perhaps only partially) the efficiency of translation, whereas protein production depends on differential mRNA abundance and perhaps many other factors. Thus, in order to evaluate the power of CAI in predicting protein production, we need at least to control for the mRNA abundance. Ideally we should have N genes all with the same mRNA abundance so that variation in protein production among these N genes can be attributed mostly to translation efficiency.

Here I use experimentally determined mRNA and protein abundance of a set of yeast (*Saccharomyces cerevisiae*) genes (Gygi et al. 1999) to evaluate the effectiveness of CAI from DAMBE (Xia and Xie, 2001) and from EMBOSS.cai, designated as DCAI and ECAI, respectively, in predicting protein production. Both DCAI and ECAI were computed by using the Eyeastcai.cut reference set. The data (Table 1) fall naturally into 11 categories of mRNA abundance, with 13 genes with mRNA abundance of 0.7, eight genes with mRNA abundance of 1.5, and so on (Table 1). An analysis of covariance, with protein abundance as the dependent variable, mRNA abundance as a categorical variable and DCAI as a covariate, results in R^2^ = 0.5421, with DCAI and mRNA abundance accounting for 37.99% and 16.22%, respectively, of the total variation in protein production, with the associated p values equal to 0.00000 and 0.0326, respectively. A similar analysis using ECAI results in R^2^ = 0.5343, with DCAI and mRNA abundance accounting for 36.05% and 17.38%, respectively, of the total variation in protein production, with the associated p values equal to 0.00000 and 0.0244, respectively. The result suggests that (1) DCAI is slightly better than ECAI, and (2) both CAI indices are better than mRNA abundance in predicting protein production within this range of mRNA abundance.

An alternative way of evaluation is simply to break the mRNA abundance into three ranges and compute the correlation between DCAI and protein abundance and between ECAI and protein abundance (Table 2). The resulting correlations also suggest that DCAI is slightly better than ECAI (Table 2). One may note that the correlation becomes much smaller in the mRNA range of 5.2–8.9 (Table 2). This is because, with substantially increased variation in mRNA abundance within this range, much more variation in protein production can be attributed to mRNA variation than to CAI variation.

## Conclusion

The improved implementation of CAI in DAMBE will help researchers to better quantify gene expression and translation efficiency of protein-coding sequences.

## Figures and Tables

**Table 1:** Data for evaluating the improved CAI from DAMBE (DCAI) and CAI from EMBOSS.cai (ECAI).

**Gene**	**SeqLen**	**DCAI**	**ECAI**	**mRNA^(1)^**	**Protein^(1)^**
APA1	966	0.452	0.405	0.7	8.7
COR1	1374	0.378	0.380	0.7	2.5
ENO1	1314	0.875	0.873	0.7	44.2
FRS2	1512	0.396	0.373	0.7	2.3
GYP6	1377	0.267	0.247	0.7	4.4
HOR2	753	0.359	0.350	0.7	5.7
IDP1	1287	0.414	0.382	0.7	7.7
PRE8	753	0.231	0.220	0.7	6.9
PUP2	783	0.268	0.226	0.7	4.4
RPE1	717	0.391	0.357	0.7	5.8
STI1	1770	0.354	0.363	0.7	13.1
TFS1	660	0.222	0.226	0.7	8.1
ZWF1	1518	0.263	0.256	0.7	5.6
ACH1	1581	0.298	0.293	1.5	9.8
ADE13	1449	0.412	0.398	1.5	6.3
CCT8	1707	0.313	0.324	1.5	2.2
PAB1	1734	0.535	0.515	1.5	30.4
PRB1	1908	0.386	0.379	1.5	21.2
SER1	1188	0.332	0.323	1.5	10.5
YEL047C	1413	0.355	0.331	1.5	3.8
YNL134C	1131	0.308	0.317	1.5	14.9
ALD6	1503	0.615	0.551	2.2	44.3
ATP1	1638	0.541	0.490	2.2	21.6
LPD1	1500	0.330	0.321	2.2	18.9
SOD2	702	0.283	0.291	2.2	12.6
TOM40	1164	0.359	0.336	2.2	22.3
YDR190C	1392	0.294	0.273	2.2	4.8
YHR049W	732	0.508	0.497	2.2	18.4
YMR226C	804	0.287	0.285	2.2	14.5
ARO8	1503	0.333	0.308	3	23.4
CAR1	1002	0.306	0.302	3	5.2
ILV6	930	0.354	0.301	3	13.9
LEU4	1860	0.394	0.368	3	3.1
PGM2	1710	0.392	0.374	3	2.2
YEL071W	1491	0.305	0.308	3	16.3
GUK1	564	0.401	0.377	3.7	16.5
IPP1	864	0.670	0.647	3.7	63.1
LYS9	1341	0.418	0.376	3.7	16.2
PRE4	801	0.250	0.257	3.7	3.4
TAL1	1008	0.641	0.586	3.7	44.8
THR4	1545	0.472	0.440	3.7	21.4
VMA4	702	0.353	0.351	3.7	10.5
YKL029C	2010	0.329	0.308	3.7	2.8
YNL010W	726	0.434	0.386	3.7	31.6
ERG10	1197	0.461	0.445	4.5	24.1
HIS1	894	0.324	0.272	4.5	22.4
HOM2	1098	0.502	0.462	4.5	60.3
ILV3	1758	0.449	0.437	4.5	5.3
ILV5	1188	0.857	0.809	4.5	76
YDL124W	939	0.282	0.277	4.5	6.4
ADE1	921	0.332	0.291	5.2	8.7
ADE3	2841	0.349	0.340	5.2	4.8
DYS1	1164	0.520	0.487	5.2	15.8
EGD2	525	0.625	0.587	5.2	20.1
GSP1	660	0.647	0.632	5.2	26.3
PRO2	1371	0.327	0.314	5.2	13.6
AAT2	1257	0.330	0.301	6	11.7
GLK1	1503	0.243	0.252	6	22.6
SEC14	915	0.390	0.365	6	10.9
URA5	681	0.379	0.365	6	25.4
YBR025C	1185	0.648	0.598	6	13.1
IDH2	1110	0.328	0.300	6.7	29.4
SPE3	882	0.450	0.425	6.7	15.1
YER067W	486	0.278	0.280	6.7	3.7
YFR044C	1446	0.378	0.342	6.7	30.2
GRS1	2004	0.464	0.450	7.4	5.5
HXK2	1461	0.681	0.664	7.4	26.5
SHM2	1410	0.677	0.629	7.4	19.7
TUB2	1374	0.342	0.337	7.4	11.2
BAT2	1131	0.274	0.257	8.9	19
CYS3	1185	0.505	0.470	8.9	6.7
URA1	945	0.314	0.288	8.9	49.5
VMA2	1554	0.455	0.437	8.9	33.7

(1) mRNA and protein abundance from Table 1 in Gygi et al. (1999), with mRNA in unit of mean copies/cell and protein in unit of 10^3^ copies/cell. Only genes that have mRNA abundance identical to at least three other genes are included.

**Table 2:** Correlation between DCAI and protein abundance (r_DCAI_) and between ECAI and protein abundance (r_ECAI_) for three ranges of mRNA abundance, with N_gene_ being the number of genes within each mRNA abundance range. Results based on data in Table 1.

mRNA range	N_gene_	^r^DCAI	^r^ECAI
0.7–2.2	29	0.7705	0.7680
3–4.5	21	0.8590	0.8376
5.2–8.9	23	0.0534	0.0478
